# Machinability Enhancement of Ti6Al7Nb Biomedical Alloy Through MWCNT-Nanofluid MQL and Vortex Tube-Assisted Side Milling

**DOI:** 10.3390/ma19173745

**Published:** 2026-09-03

**Authors:** Aqib Mashood Khan, Barlas Gökduman, Erkin Duman, Hasan H. Hijji, Yusuf Furkan Yapan, Muhammad Ahmed Khan, Adnan Javed, Alper Uysal

**Affiliations:** 1 College of Mechanical and Electrical Engineering, Nanjing University of Aeronautics and Astronautics, Nanjing 210016, China; dr.aqib@nuaa.edu.cn (A.M.K.); adnanjaved@nuaa.edu.cn (A.J.); 2 Department of Mechanical Engineering, Yildiz Technical University, Besiktas, Istanbul 34349, Türkiyeyapan@yildiz.edu.tr (Y.F.Y.); 3 Gedik Vocational School, Department of Machine and Metal Technology, Istanbul Gedik University, Istanbul 34913, Türkiye; erkin.duman@gedik.edu.tr; 4 Mechanical Engineering Department, College of Engineering and Architecture, Umm Al-Qura University, P.O. Box 5555, Makkah 21955, Saudi Arabia; 5 School of Mechanical Engineering, Shandong University, Jinan 250061, China; ahmedkhan@mail.sdu.edu.cn

**Keywords:** side milling, multi-walled carbon nanotubes, vortex tube cooling, cutting forces, machined surface quality

## Abstract

Ti6Al7Nb biomedical alloy is difficult to machine because its low thermal conductivity and high chemical reactivity promote heat accumulation, high cutting loads, and poor surface quality. This study evaluated the side milling performance of Ti6Al7Nb under dry machining, vortex tube cooling, and MWCNT-assisted nanoparticle minimum quantity lubrication (NMQL) to identify a more sustainable and effective machining strategy. Experiments were conducted using two cutting speeds and three feed rates, and machinability was assessed in terms of cutting temperature, resultant cutting force, surface roughness, Tol wear and tool life, chip morphology, and multi-criteria ranking. Compared with dry machining, vortex tube cooling provided the strongest thermal control, reducing cutting temperature by 25–36% compared with dry conditions, owing to the cold air stream generated by the Ranque–Hilsch effect. MWCNT-NMQL produced the greatest reductions in cutting force and surface roughness, with improvements of 7–28% and 10–20%, respectively, compared with dry conditions, due to improved lubrication, reduced adhesion, possible tribofilm formation, rolling/sliding effects of MWCNTs, and enhanced heat transfer. Chip morphology observations confirmed that both assisted environments improved chip formation compared with dry machining. The overall ranking identified vortex tube cooling at Vc = 30 m/min and f = 0.08 mm/rev as the best overall condition, while NMQL was more favorable for force reduction and surface finish improvement. The findings of this study provide practical guidance for the selection of sustainable and effective cutting strategies in the precision machining of biomedical titanium alloys.

## 1. Introduction

Machining performance in modern manufacturing is strongly influenced by the selection of process parameters, as these directly affect productivity, cost, and product quality [1]. During the material removal process, a significant portion of mechanical energy is transformed into heat, leading to a rise in temperature within the cutting zone [2]. The extent of this temperature increase depends on both the workpiece material and the applied cutting conditions, including spindle speed, feed rate, and depth of cut [3]. Excessive heat generation is widely recognized as one of the main causes of machining-related problems since it can adversely affect tool life, dimensional accuracy, and surface quality. Consequently, effective cooling and lubrication strategies are essential for achieving stable and efficient machining performance [4]. Over the past two decades, various cooling approaches have been explored; however, growing economic pressures and environmental concerns have increased the demand for more sustainable machining practices [5]. In this context, international environmental frameworks such as ISO 14001 have further encouraged the adoption of cleaner and more responsible manufacturing methods [6].

Titanium alloys are widely recognized as essential engineering materials in aerospace, biomedical, marine, and chemical industries because of their high strength-to-weight ratio, strong corrosion resistance, and good biocompatibility [7]. Among these alloys, Ti6Al7Nb has attracted particular interest in biomedical applications as a safer alternative to the commonly used Ti-6Al-4V alloy, mainly because it eliminates vanadium, which has been associated with possible toxic effects in the human body [8]. The replacement of vanadium with niobium improves the biological acceptance of the alloy while preserving its desirable mechanical performance [9]. As a result, Ti6Al7Nb has become a promising material for orthopedic implants, dental components, and cardiovascular devices, where both mechanical stability and long-term compatibility with body tissues are essential [10]. However, despite these advantages, Ti6Al7Nb remains a difficult-to-machine material. Its machining is often accompanied by high cutting temperatures, rapid tool wear, and compromised surface quality, which can limit production efficiency and increase manufacturing cost [11]. These challenges make it necessary to explore advanced cooling and lubrication approaches that can support more efficient, sustainable, and economically practical machining processes.

Although conventional flood cooling has long been used in machining operations, recent research has increasingly shifted toward more efficient and economical alternatives such as minimum quantity lubrication (MQL). A considerable number of studies have examined the influence of MQL on machining performance [12,13,14,15]. For example, Attanasio et al. [16] reported that, during the turning of 100Cr6 steel, MQL substantially reduced flank wear, although its effect on rake face wear was limited. In the finish turning of Inconel 718, Kamata and Obikawa showed that increasing the cutting fluid flow rate under MQL improved tool life and surface finish when TiAlN-coated carbide tools were used, whereas TiN/AlN PVD-coated tools mainly exhibited improvements in surface finish rather than tool life [17]. Several other researchers have also highlighted the effectiveness of MQL, particularly at relatively low cutting speeds, although some studies have demonstrated satisfactory performance even at higher cutting speeds. In the high-speed milling of aluminum alloys, Kishawy et al. [18] found that both MQL and flood cooling reduced cutting forces compared with dry machining, mainly because the presence of fluid decreased adhesion and friction at the cutting interface. Likewise, Rahim et al. [19] observed that, in drilling Ti-6Al-4V, MQL provided longer tool life than dry machining under all tested conditions, primarily due to lower friction, reduced temperature, and less severe tool chipping. They further noted that vegetable-based lubricants such as palm oil could serve as promising alternatives because of their favorable effects on tool life, torque, and cutting force [20]. While it is widely regarded as an environmentally friendly lubrication strategy, its cooling capability is often lower than that of conventional flood cooling.

The quantity of cutting fluid used in MQL systems is often insufficient to provide the desired cooling and lubrication effects for many machining applications [21]. As a result, improving the performance of MQL systems by integrating additional techniques is essential. One promising approach is the incorporation of nanoparticles into the base fluid [22]. The addition of nanoparticles, such as graphite, aluminum oxide (Al_2_O_3_), molybdenum disulfide (MoS_2_), hexagonal boron nitride (hBN), graphene, carbon nanotubes, and multi-walled carbon nanotubes (MWCNTs), has been shown to enhance the thermal conductivity of the fluid, improving its overall efficiency [23]. Furthermore, nanoparticles contribute to better tribological performance by reducing tool wear and the coefficient of friction during machining. The addition of even small volumes of nanoparticles significantly increases the surface area of the cooling medium, enhancing heat transfer and enabling stable Brownian motion of the particles [24]. Studies have demonstrated that MWCNT-assisted MQL can provide significant improvements in machining performance, particularly in reducing surface roughness. This effect is attributed to the formation of a stable tribological thin film and the filling of micro-pores on both the machined surface and tool cutting edge, which leads to better lubrication and smoother cutting conditions [25]. In one study, the performance of silver and alumina nanoparticles was compared for MQL during the machining of nickel alloys. Alumina nanoparticles contributed to reduced cutting force, wear, and chip curling, owing to their fine droplet formation and enhanced particle spread ability. Silver nanoparticles exhibited excellent ball-bearing effects, resulting in improved surface quality and reduced abrasive wear [26]. Recent studies have explored the use of Al_2_O_3_-MWCNT hybrid nanofluids to assess their impact on various machining characteristics, including temperature control, surface finish, and tool life, particularly when machining hard materials. These hybrid nanofluids were prepared by dispersing the nanoparticles in vegetable oil, and the results showed significant improvements compared with traditional cooling methods, such as cryogenic CO_2_ cooling. Specifically, the application of these hybrid nanofluids resulted in an 8.72% improvement in surface finish, an 11.8% reduction in cutting force, and a 23% increase in tool life [27]. In another study, Zhang et al. [28] applied MoS_2_-MWCNTs as additives in grinding operations and observed a reduction in the grinding ratio and surface roughness compared with using MoS_2_ or MWCNTs individually. In the context of MQL, MWCNT-based nanofluids dispersed in vegetable oil were utilized in the machining of titanium alloys at varying concentrations to optimize the turning process. The study revealed that a 2 wt% concentration of MWCNTs led to an 11.5% reduction in power consumption compared with standard MQL without additives [29]. This demonstrates the efficiency of MWCNT nanofluids in reducing energy consumption while maintaining or improving machining performance.

To enhance the thermal and tribological properties of nanofluids, the application of Al_2_O_3_-MWCNT hybrid nanoparticles has been studied across different concentrations. The findings from these studies showed a significant reduction in wear and a lower coefficient of friction, which can be attributed to the superior lubrication, improved wettability, and effective dispersion of the nanoparticles in the fluid. Specifically, hybrid nanofluids resulted in 11% less flank wear and a 27.36% reduction in nodal temperature when compared with Al_2_O_3_-based MQL systems. The differing morphologies of the Al_2_O_3_ and MWCNT nanoparticles, spherical and cylindrical, respectively, contribute to their ability to penetrate the machining surface at different depths, improving overall lubrication efficiency [30]. Furthermore, the unique properties of MWCNTs play a key role in reducing friction. Their relatively weak van der Waals forces allow for easy shearing under external loads, facilitating inter-tubular slip at the tool–chip interface. The high thermal conductivity of MWCNTs (ranging from 3000 to 6000 W/m°C) helps dissipate heat efficiently, while the formation of a tribological layer on the workpiece surface further reduces friction over time. This tribo-layer effect enhances the long-term performance of the machining process by continuously lowering friction, which in turn reduces wear and extends tool life [31].

Recent research has focused on combining vortex tube cooling (RHVT) with MQL to overcome MQL’s insufficient cooling capacity while maintaining its lubrication and environmental benefits. Singh et al. explored this hybrid approach by integrating RHVT into the MQL delivery system during the turning of commercially pure titanium. The cold air from the vortex tube chilled the oil mist before it reached the cutting zone, which led to lower cutting temperatures and better surface quality compared with MQL alone. This suggests that the vortex tube’s main role is thermal management rather than improving lubrication [7]. Meanwhile, Dongre et al. [32] investigated the turning of Ti-6Al-4V under various cooling conditions, including dry, MQL, cryogenic, and vortex jet cutting. Their study compared the performance of uncoated carbide, TiN-coated, and Al_2_O_3_-coated inserts. They concluded that vortex jet cooling should be considered a distinct and independent cooling method not just an auxiliary to other techniques like cryogenic cooling or MQL. Despite this, vortex tube cooling is often still used as a secondary method, which presents a gap in machining research. The simplicity, low cost, and zero chemical waste nature of RHVT cooling make it a promising candidate for future research, particularly for Ti6Al7Nb alloys, where machining data under advanced cooling methods remain scarce [8]. Compared with the extensive literature on Ti6Al4V, studies focusing on Ti6Al7Nb machining remain limited, although this alloy is highly relevant for biomedical implants. Sun et al. [8] showed that a cooling strategy strongly affects the surface quality of Ti6Al7Nb, while Sharma et al. [33] emphasized the importance of surface quality, bioactivity, and performance characteristics of machined Ti6Al7Nb biomedical alloy. In addition, sustainable machining reviews highlight the need for advanced cooling/lubrication strategies when machining difficult-to-machine titanium alloys [34]. Therefore, the present work strengthens this research direction by experimentally comparing MWCNT-based NMQL and vortex tube cooling during side milling of Ti6Al7Nb biomedical alloy. Ti6Al7Nb is widely used in biomedical components such as orthopedic and dental implants; therefore, machining results should be considered from a surface integrity perspective. Surface characteristics strongly influence implant performance, as excessive heat, deformation, irregular topography, defects, and contamination can reduce reliability.

Building on this research gap, the present study directly compares two cleaner machining strategies—vortex tube cooling and MWCNT-based NMQL—for side milling of Ti6Al7Nb under identical cutting parameters. Compared with Ti6Al4V, Ti6Al7Nb has received considerably less attention in milling studies despite its biomedical relevance. The study distinguishes the thermal control performance of vortex tube cooling from the lubrication and surface quality performance of MWCNT-based NMQL by evaluating cutting temperature, resultant cutting force, surface roughness, tool wear and tool life, chip morphology, and multi-criteria decision-making rankings. The MWCNT-related mechanisms discussed in this work are considered plausible explanations based on the previous literature rather than mechanisms directly characterized in the present experiments. Furthermore, because residual stress, microstructure, microhardness, and surface chemistry were not investigated, the measured responses should be considered machinability and surface-related parameters relevant to surface integrity rather than a comprehensive surface integrity assessment. The comparison with previous studies and the specific research gap addressed by the present work are summarized in Table 1. These insights are particularly relevant for industries such as aerospace and biomedical applications, where Ti6Al7Nb is commonly used and where thermal control and surface quality are critical.

## 2. Experimental Procedures

### 2.1. Materials, Cutting Tools, and Machine Tool

Ti6Al7Nb alloy was employed as the work material, with initial dimensions of 36 mm in diameter and a thickness of 2.5 mm. A side milling experiment was conducted. The FIRST MCV-300 model CNC machining center (Long Chang Machinery Co., Ltd., Taichung, Taiwan) and cutting tool were selected, then the Karcan 155508010 Ø8 X-PER AlCrN-coated carbide tool (Karcan Cutting Tools, Eskişehir, Türkiye). Cutting parameters included two cutting speeds (Vc), and three feed values *f* (linear tool feed per spindle revolution and is expressed in mm/rev). Three distinct cutting conditions were applied during the experiments: dry, N-MQL, and vortex tube cooling. The radial depth of cut was kept constant at 0.5 mm. A full factorial experimental design was used, consisting of 18 experimental combinations obtained from two levels of cutting speed, three levels of feed rate, and three cutting environments, as shown in Table 2. To ensure the repeatability and reliability of the results, each experimental condition was repeated three times under identical machining parameters. The cutting temperature, resultant cutting force, and surface roughness results are presented as the arithmetic mean of the three repetitions. The variability of the measurements was evaluated using standard deviations, and the corresponding error bars were included in the result figures. In addition, analysis of variance (ANOVA) was performed to determine the statistical significance and contribution of the investigated process parameters to each response.

For statistical modeling, the three cutting environments were coded as Dry = −1, Vortex = 0, and NMQL = +1. These coded values were used solely as a mathematical representation of the three experimental levels and do not represent a continuous physical scale. Accordingly, the first- and second-order terms associated with cutting condition were interpreted as statistical contrasts among the three environments rather than as continuous linear and nonlinear physical effects.

**Figure 1:**
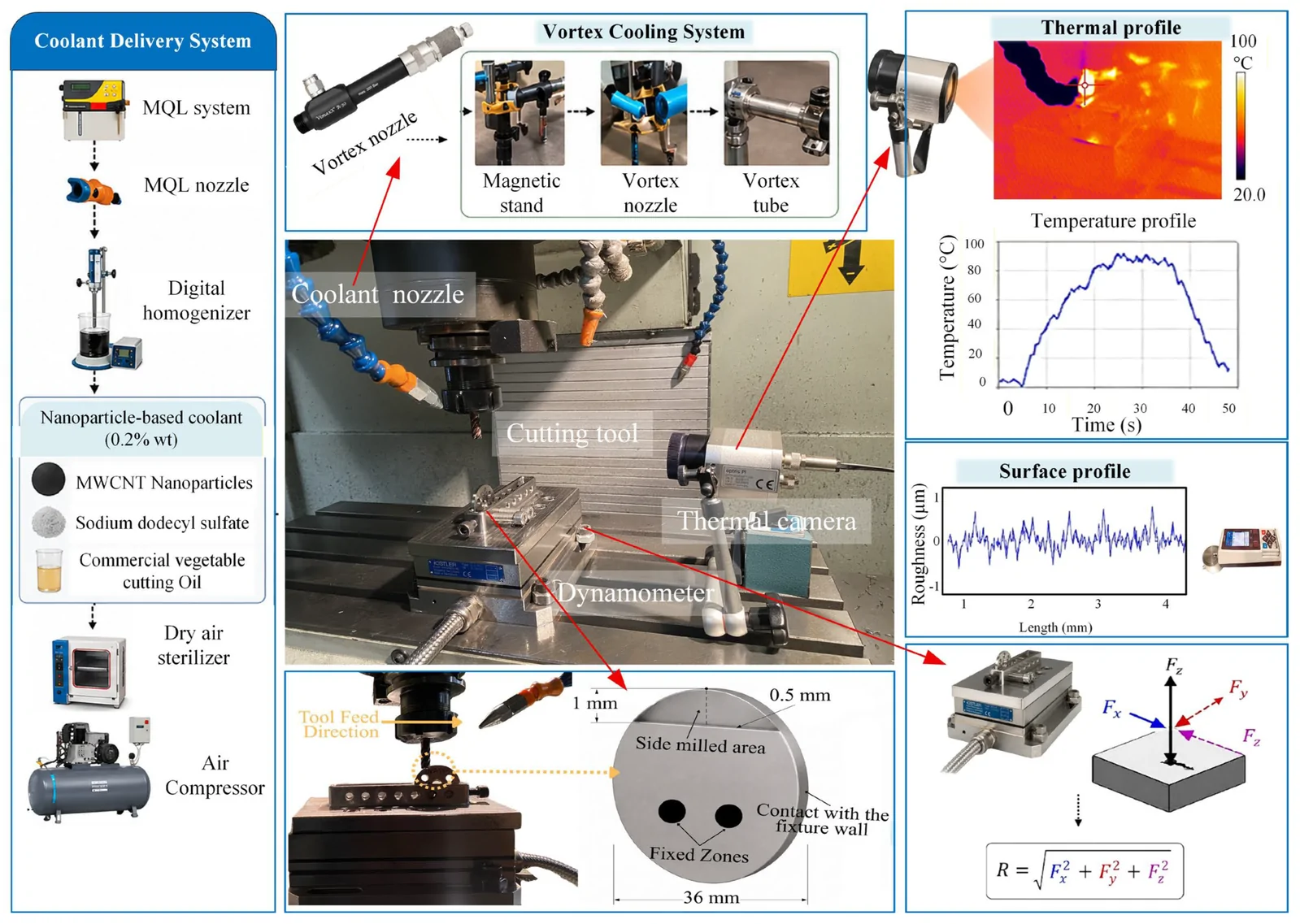
Experimental setup for side milling of Ti6Al7Nb using N-MQL and vortex tube cooling.

In the N-MQL setup, a multi-walled carbon nanotube (MWCNT)-based nanofluid was delivered to the cutting area. The nanofluid preparation process involved drying the MWCNT particles and subsequently dispersing them into the cutting oil at a concentration of 0.2 wt%. To further enhance thermal management during machining, air cooling was applied via a vortex tube. During the milling operations, cutting forces and surface roughness were measured using a Kistler 9257BA dynamometer (Kistler Instrumente AG, Winterthur, Switzerland) and a Mitutoyo SJ-210 surface roughness tester (Mitutoyo Corporation, Kawasaki, Kanagawa, Japan), respectively. The Ti6Al7Nb disc was mounted in a rigid custom fixture fixed directly on the Kistler 9257BA dynamometer to reduce possible elastic deflection and vibration during side milling. Although the workpiece thickness was 2.5 mm, the operation was not considered thin wall milling because the disc was not machined as a free-standing flexible wall. The specimen was supported from the bottom and clamped over a large contact area, while only the peripheral region required for material removal was exposed to the cutting tool. This fixture configuration increased the stiffness of the workpiece–fixture system and helped maintain cutting stability during force, temperature, and surface roughness measurements. Therefore, the machining operation was defined as side milling. An Optris PI thermal camera (Optris GmbH & Co. KG, Berlin, Germany) was used to measure the cutting temperature non-contactly during machining of the Ti-6Al-7Nb alloy. The temperature variations in the cutting zone were monitored and recorded within the 0–250 °C measurement range, with a thermal image resolution of 382 × 288 pixels and a frame rate of 80 Hz. The thermal camera was positioned at a fixed distance of approximately 30 cm from the cutting zone to obtain a clear and stable view of the machining area. Since the emissivity of metallic surfaces can significantly affect infrared temperature measurements, the emissivity value was calibrated before the experiments. For this purpose, the Ti-6Al-7Nb workpiece material was heated under controlled conditions, and the temperature was measured simultaneously using both the thermal camera and a thermocouple. The emissivity value in the camera software was then adjusted until the camera temperature matched the thermocouple reading. Based on this calibration procedure, the emissivity value was set to 0.63 for Ti-6Al-7Nb. During the experiments, the camera was fixed to directly observe the cutting zone. The region of interest was selected as the tool–workpiece interaction area, where the highest cutting temperatures occurred. The radiometric thermal images were recorded using PI Connect software (version 3.14.3086.0), and the temperature–time data were analyzed to determine the maximum cutting temperature and its variation during machining. Chip morphology was examined with an Mshot model microscope (Guangzhou Micro-shot Optical Technology Co., Ltd., Guangzhou, Guangdong, China). The experimental setup is illustrated in Figure 1, and additional details regarding the experimental conditions and parameters are presented in Table 3.

### 2.2. Preparation Process of MWCNT Nanoparticles and Mixing with MQL

The cutting fluid employed in the experiments was the commercial vegetable-based lubricant Eraoil KT/2000 (Werte, İstanbul, Türkiye), which had a density of 0.85 g/cm^3^ at 20 °C, a viscosity of 11 cst at 20 °C and a reddish-orange color. Eraoil KT/2000 was used without dilution with water; therefore, the prepared nanofluid should be considered an oil-based MWCNT suspension rather than a water-soluble cutting fluid formulation. The multi-walled carbon nanotubes (MWCNTs) used were from the NANOCYL™ NC7000 series (Nanocyl SA, Sambreville, Belgium), which were purified to 90%, had an average diameter of 9.5 nm, a length of 1.5 μm, a surface area of 250–300 m^2^/g and a thermal conductivity of about 3000 W/m·K.

Before preparation of the nanofluid, the MWCNT nanoparticles were dried in an oven at 120 °C for 2 h to remove possible moisture. After drying, the nanoparticles were weighed using a high-precision Radwag PS 510 digital balance and then dispersed into the base oil at a concentration of 0.2 wt.%. Sodium dodecyl sulfate (SDS) was added as a surfactant at 0.1 wt.% relative to the MWCNT content to support nanoparticle dispersion, homogeneity, and suspension stability. Although SDS is commonly used in aqueous systems due to its hydrophilic character, in the present study it was used based on the lubricant manufacturer’s recommendation and the authors’ previous experience with NMQL applications [34,35]. Therefore, SDS was employed as a dispersion-supporting additive together with intensive mechanical and ultrasonic mixing, rather than as an independently optimized surfactant system for long-term oil-based nanofluid stability.

The mixture was mechanically homogenized using a Daihan HG-15D digital homogenizer (DAIHAN Scientific Co., Ltd., Wonju-si, Republic of Korea) at 5000 rpm for 1 h. Subsequently, ultrasonic homogenization was performed using a WEIGHTLAB WU-6 ultrasonic bath (Weightlab Instruments, İstanbul, Türkiye) at 20 kHz and 200 W for 1 h to break down possible MWCNT agglomerates and improve the initial dispersion quality. Finally, the suspension was stirred using a WEIGHTLAB WN-H320 magnetic stirrer (Weightlab Instruments, İstanbul, Türkiye) at 1500 rpm for 1 h to further homogenize the nanoparticle distribution. The prepared nanofluid was used immediately after preparation, and no visible sedimentation was observed before the start of the NMQL cutting tests. To maintain homogeneity during the entire machining process, a custom-designed mechanical mixer with adjustable speed was integrated into the MQL system (Werte, İstanbul, Türkiye). This mixer ensured that the nanofluid was continuously agitated, thereby preventing sedimentation and maintaining a uniform distribution of nanoparticles in the cutting fluid. Each experimental condition was repeated three times under the same cutting parameters and environment. The reported values of cutting temperature, cutting force, and surface roughness represent the averages of these trials. Standard deviations evaluated variability, with error bars added to figures. For cutting force, the resultant force was calculated from measured components during stable cutting. For temperature, the maximum/average zone temperature was extracted from thermal camera recordings. For roughness, measurements at three locations on each surface were averaged. Since a conventional MQL condition without MWCNTs was not included, the improvements observed under MWCNT-NMQL should be interpreted as the combined effect of the base oil mist and possible nanoparticle-assisted lubrication/heat transfer mechanisms rather than as the isolated effect of MWCNTs alone.

## 3. Results and Discussion

### 3.1. Cutting Temperature Results

**Figure 2:**
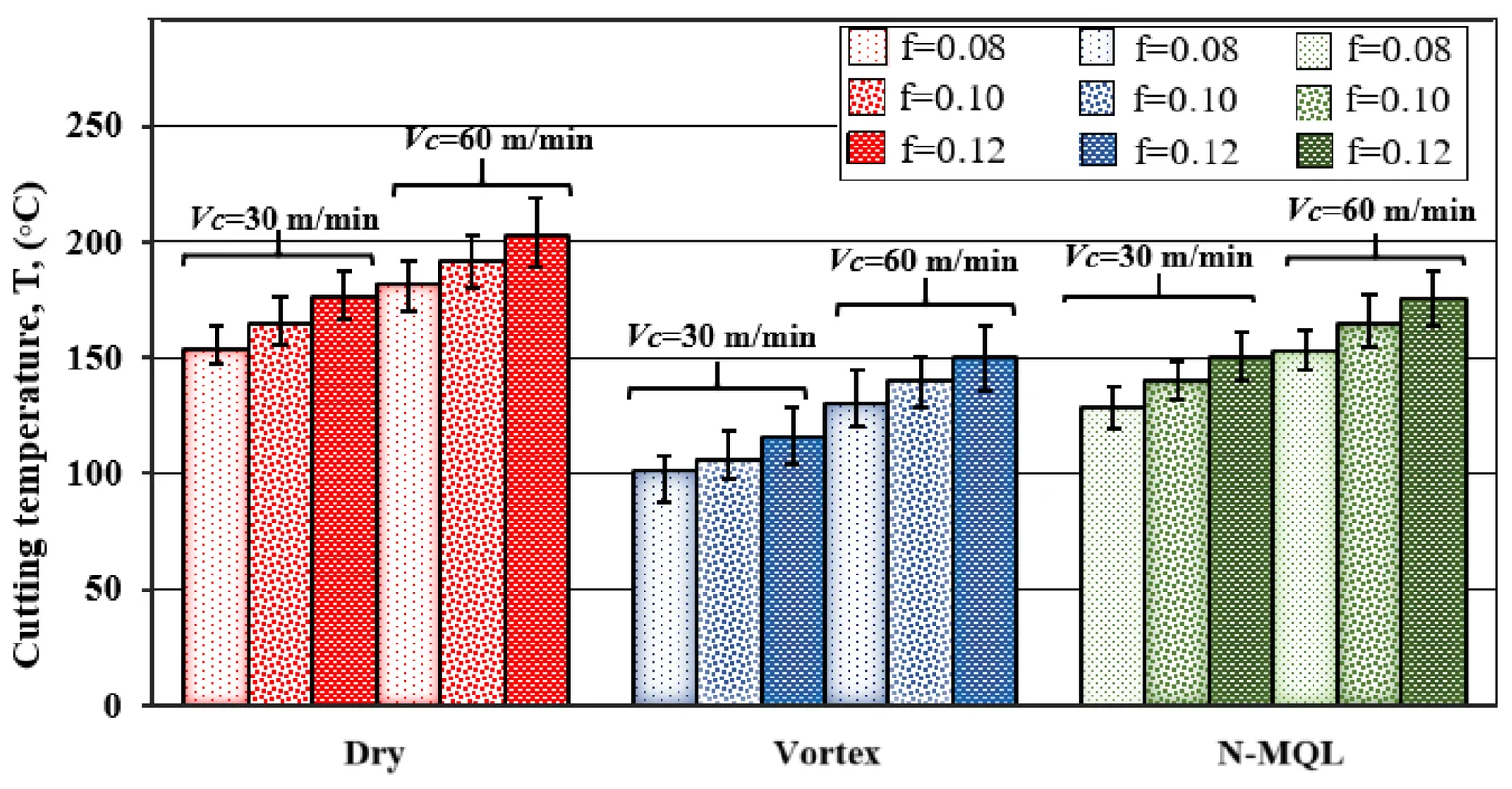
Cutting zone temperature response to varying feed rates and cutting environments during side milling of Ti6Al7Nb at V_c_ = 30 and 60 m/min under dry, vortex tube, and NMQL conditions.

Figure 2 shows that the cutting temperature increased with both cutting speed and feed rate under all machining conditions. This trend is expected because a higher cutting speed increases the relative sliding velocity at the tool–chip interface, while a higher feed rate increases the undeformed chip thickness. Both effects increase plastic deformation, frictional contact, and heat generation in the cutting zone.

Among the three cutting environments, dry machining produced the highest cutting temperatures. At *V_c_* = 30 m/min, the dry condition generated temperatures of approximately 155–178 °C, while at *V_c_* = 60 m/min, the temperature increased to nearly 182–203 °C. This is mainly because no external cooling or lubrication was available in dry cutting. Therefore, most of the heat generated by plastic deformation and tool–chip friction remained concentrated near the cutting edge. The vortex tube condition showed the lowest cutting temperatures for all cutting parameters. At *V_c_* = 30 m/min, the temperature was reduced to around 100–116 °C, and at *V_c_* = 60 m/min, it increased only to about 130–150 °C. This confirms the strong thermal control ability of vortex cooling. Physically, the cold compressed air from the vortex tube removes heat from the cutting zone through forced convection. Since titanium alloys have low thermal conductivity, heat does not dissipate easily into the workpiece. Therefore, external cold air cooling becomes highly effective in reducing the localized temperature around the tool–chip interface.

The NMQL condition also reduced cutting temperature compared with dry machining, but its cooling effect was lower than that of vortex cooling. Under NMQL the temperature ranged from approximately 128–151 °C at *V_c_* = 30 m/min to 154–176 °C at *V_c_* = 60 m/min. This improvement can be attributed to the combined action of the cutting oil and MWCNT nanoparticles [8,34]. As reported by Aydın et al. [34], the oil reduces friction at the tool–chip interface, while the MWCNTs enhance the thermal conductivity of the lubricant and improve heat transfer. However, because MQL supplies only a small quantity of fluid, its cooling capacity remains limited compared with the direct cold air stream produced by the vortex tube. Overall, the results indicate that vortex cooling is the most effective method for reducing cutting temperature while NMQL provides moderate thermal improvement compared with dry machining. The lowest temperature was obtained under vortex cooling at the lower cutting speed and lowest feed rate. This suggests that for Ti6Al7Nb side milling, temperature control is governed mainly by the ability of the cooling method to remove heat from the cutting zone rather than by lubrication alone. High cutting temperatures in dry machining suggest a greater risk of thermal damage, including adhesion, oxidation, and microstructural changes. In contrast, vortex tube cooling significantly reduces temperature, indicating lower thermal impact and improved surface stability.

Table 4 presents the ANOVA results for cutting temperature. Cutting condition was identified as the most influential factor, with a contribution ratio of 64.83%, followed by cutting speed and feed rate, with contribution ratios of 25.76% and 8.92%, respectively. All three main factors were statistically significant at the 95% confidence level (*p* < 0.05). In contrast, the second-order terms and two-way interaction terms were statistically non-significant. The second-order component associated with cutting condition contributed only 0.01% and was statistically non-significant (*p* = 0.585), indicating that the temperature response at the intermediate coded level did not show a statistically meaningful deviation relative to the two outer coded levels. Among the interaction terms, the interaction between cutting speed and cutting condition showed the highest contribution, although its effect remained statistically non-significant. The total error contribution was only 0.27%, indicating that the developed ANOVA model explained nearly all of the variability in the cutting temperature results. These findings demonstrate that the cutting environment is the dominant parameter controlling cutting temperature, while cutting speed also has a considerable effect. In particular, vortex tube cooling produced the lowest cutting temperatures across the investigated parameter range, whereas dry machining resulted in the highest values.

### 3.2. Cutting Force

**Figure 3:**
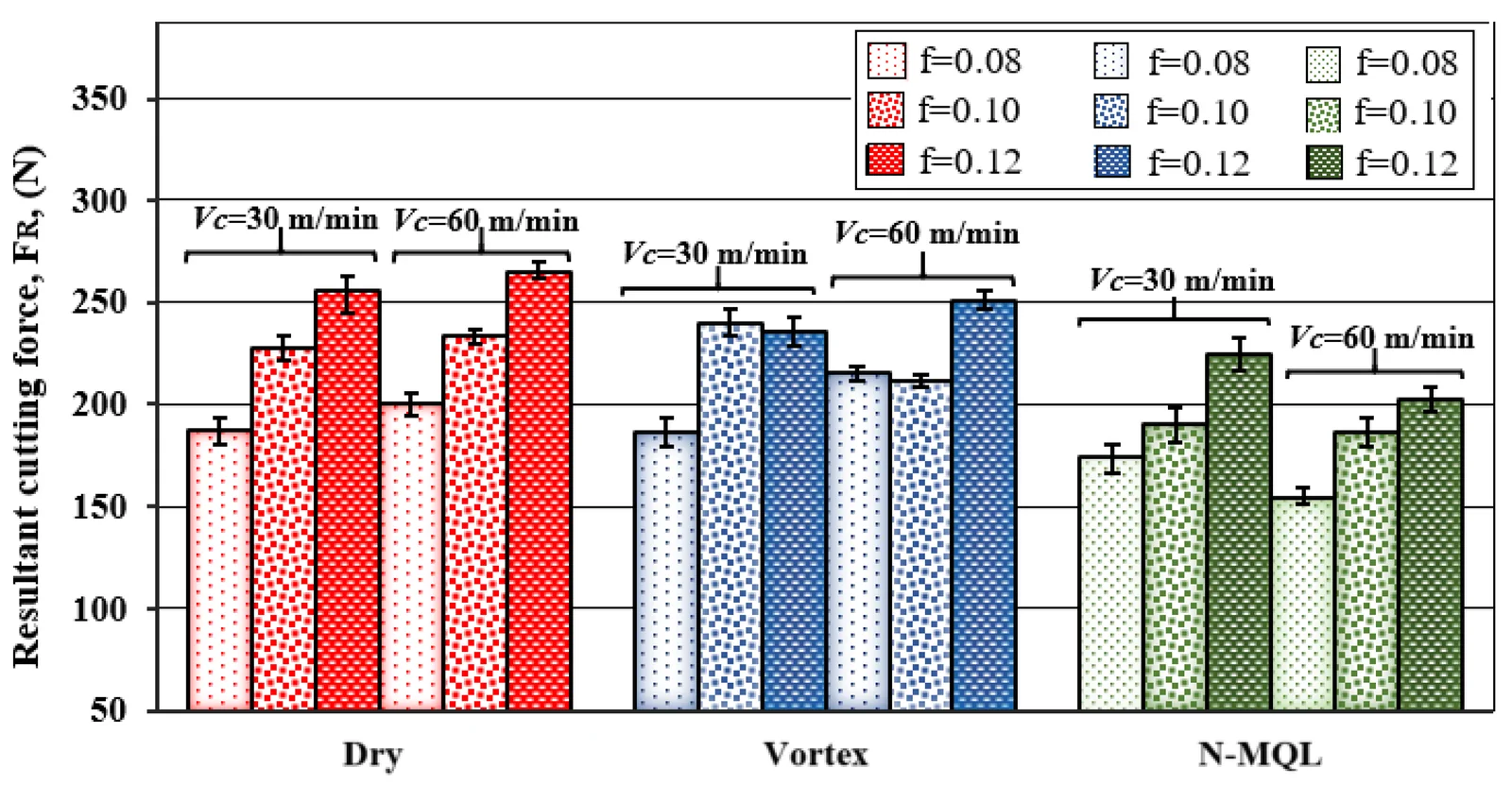
Effect of feed rate and diverse cutting environment on resultant cutting force during side milling of Ti6Al7Nb.

Figure 3 illustrates the resultant cutting force recorded during side milling of the workpiece across three cutting environments—dry, vortex tube cooling, and nanoparticle-enriched MQL—at two cutting speeds 30 and 60 m/min and three feed rates (f = 0.08, 0.10, and 0.12 mm/rev), with a constant depth of cut of 1 mm and radial depth of cut of 0.5 mm.

Across all three cutting environments and both cutting speeds, the resultant cutting force exhibited a consistent and monotonically increasing trend with rising feed rate. At f = 0.08 mm/rev, the lowest resultant force values were observed in each condition, whereas at f = 0.12 mm/rev, the highest forces were recorded. This behavior is physically well-grounded, an increase in feed rate directly enlarges the uncut chip cross-sectional area, which amplifies the mechanical load that the cutting tool must overcome to shear the workpiece material. A greater chip thickness requires a larger deformation force in the primary shear zone and the consequent increase in the tool–chip contact area intensifies friction along the rake face, both of which contribute additively to the rise in the resultant cutting force. This relationship aligns with the classical chip formation mechanics described by Merchant’s cutting force model and has been consistently reported in the milling literature for a wide range of workpiece materials.

The dry condition produced the highest resultant cutting forces overall, reaching approximately 265 N at f = 0.12 mm/rev and *V_c_* = 30 m/min. The absence of any cooling or lubricating medium means that the full frictional resistance at the tool–chip and tool–workpiece interfaces must be overcome mechanically. Elevated interfacial temperatures in dry cutting promote adhesion between the chip and rake face, increasing the chip contact length and frictional force component. The vortex tube condition yielded intermediate cutting force values generally lower than dry machining but higher than NMQL. The cold air stream generated by the vortex tube effectively reduces the cutting zone temperature and suppresses thermal softening of the tool. However, because the vortex tube delivers only pneumatic cooling without any lubricating film, the coefficient of friction at the tool–chip interface remains relatively high. This explains why, despite its thermal benefit, the vortex tube cannot achieve the force reductions offered by a lubricated environment. The NMQL condition consistently produced the lowest resultant cutting forces across all parametric combinations. This performance may be associated with the combined lubricating effect of the oil mist and possible nanoparticle-assisted tribological and thermal mechanisms reported in previous studies [34]. The base oil provides a continuous lubricating film at the tool–chip interface, significantly reducing the coefficient of friction and the adhesion component of the cutting force. The MWCNT nanoparticles suspended in Eraoil KT/2000 further amplify the lubricating effectiveness through two distinct mechanisms reported in previous studies. First, the nanotubes physically interpose between contacting asperities at the tool–chip interface, possibly acting as nanoscale rolling elements that reduce direct metallic contact and lower sliding friction [34]. In this context, these mechanisms are presented as plausible explanations supported by the existing literature, while the present work primarily focuses on evaluating the overall machining performance improvements. Second, owing to their exceptionally high axial thermal conductivity, MWCNTs may support heat transfer away from the cutting zone, preventing thermal buildup that would otherwise accelerate tool wear and promote workpiece surface damage [35]. These two mechanisms act concurrently, reducing shear stress in both the primary and secondary deformation zones. Consequently, NMQL produced the lowest resultant force values among all tested environments, demonstrating the clear tribological advantage of nanoparticle-enriched lubricants in side milling operations.

The influence of cutting speed on resultant force presented a noteworthy trend that, in the dry and NMQL conditions, forces at *V_c_* = 60 m/min were generally lower than those at *V_c_* = 30 m/min for equivalent feed rate settings, while, in the vortex tube condition, an opposite tendency was observed at f = 0.12 mm/rev. The reduction in cutting force with increasing speed under dry and NMQL conditions can be attributed to the thermal softening effect that accompanies elevated cutting temperatures at higher speeds. As cutting speed increases, the rate of heat generation in the shear zone rises and the resulting temperature elevation lowers the shear strength of the workpiece material thereby reducing the force required for chip separation. Additionally, higher cutting speeds promote a more favorable chip morphology and reduce the built-up edge (BUE) tendency on the tool rake face, which would otherwise increase the effective cutting angle and elevate force components. The slightly anomalous rise observed in vortex tube cooling at an elevated feed and speed can be linked to the aggressive cooling effect of the cold air jet combined with the added ploughing forces that arise when chip thickness increases simultaneously.

Table 5 presents the corresponding ANOVA results. The feed rate was identified as the most influential factor, with a contribution ratio of 52.76%, and its effect was statistically significant (*p* < 0.001). This result agrees with the experimental trend, in which the resultant cutting force generally increased with an increasing feed rate because of the larger undeformed chip thickness and higher mechanical load on the cutting edge. The second-order component associated with the coded cutting condition was also statistically significant (*p* < 0.001) and accounted for 34.68% of the total variation. Under the adopted −1, 0, and +1 coding, this significant component indicates that the resultant cutting force response obtained under the intermediate coded condition, vortex, deviated significantly from the response pattern represented by the two outer coded conditions, dry and NMQL. Importantly, this term should be interpreted as a statistical contrast among the three cutting environments and not as evidence of a physically nonlinear dependence of cutting force on the cutting environment. The first-order component associated with cutting condition contributed only 0.46% and was statistically non-significant (*p* = 0.532). This indicates that the direct contrast represented by the first-order coded component was relatively small compared with the second-order contrast. Nevertheless, the experimental results clearly showed differences among the three cutting environments, with NMQL generally producing the lowest resultant cutting forces, while dry and vortex tube conditions exhibited comparatively higher force levels depending on the selected feed rate and cutting speed. Cutting speed had a negligible contribution and was statistically non-significant (*p* = 0.969). Similarly, the second-order feed term and all two-way interaction terms were statistically non-significant (*p* > 0.05). The error contribution was 9.81%, indicating that the ANOVA model explained approximately 90.19% of the total variability in the resultant cutting force data. Overall, the results demonstrate that the feed rate was the dominant parameter affecting the resultant cutting force, while the cutting environment also had a substantial influence, particularly through the statistically significant contrast represented by its second-order coded component.

### 3.3. Surface Roughness (R_a_)

**Figure 4:**
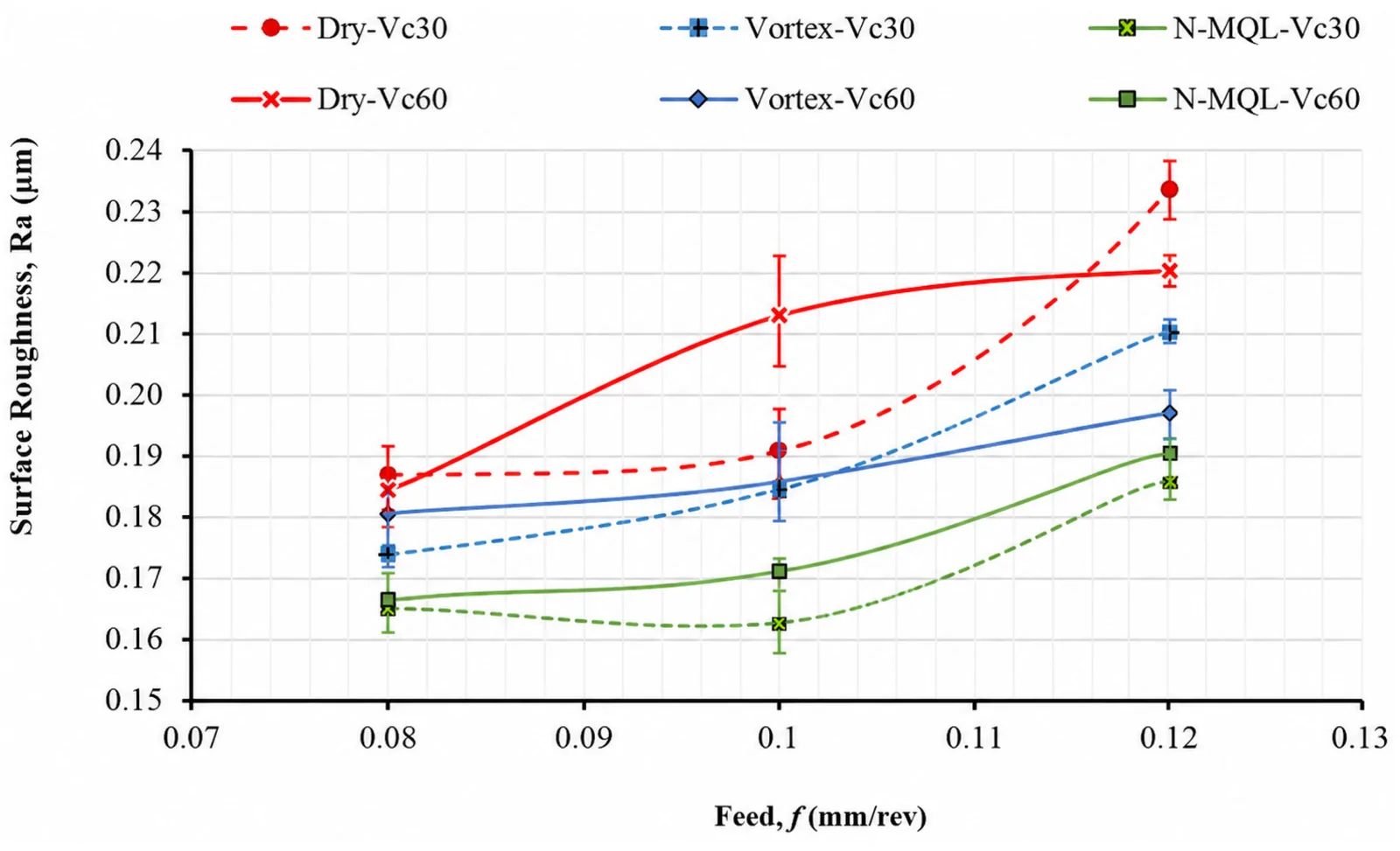
Variation of surface roughness (Ra) with feed rate at two cutting speeds under dry, vortex tube cooling, and NMQL environments during side milling of Ti6Al7Nb.

Figure 4 presents the variation in surface roughness (Ra) as a function of feed rate across three cutting environments—dry, vortex tube cooling, and NMQL—at cutting speeds of *V_c_* = 30 m/min and 60 m/min.

A clear and consistent increase in surface roughness was observed with a rising feed rate across all cutting conditions. The lowest Ra values were recorded at f = 0.08 mm/rev, while the highest were obtained at f = 0.12 mm/rev, regardless of the cutting environment or speed. As the feed rate increases, the theoretical peak-to-valley height of the feed marks left on the machined surface becomes larger since the tool traverses a greater distance per revolution, leaving a coarser scallop profile. Beyond geometric considerations, a higher feed rate also intensifies the mechanical load on the cutting edge promoting micro-vibrations and chatter tendencies that further deteriorate the surface finish. Additionally, increased chip thickness at higher feed rates elevates the frictional heat at the tool–workpiece interface, which can induce localized material smearing and plastic side flow, both of which contribute to a rougher surface texture.

The influence of cutting speed on Ra varied depending on the cutting environment. In dry machining, *V_c_* = 60 m/min produced noticeably higher Ra than *V_c_* = 30 m/min. This deterioration is attributed to the intensified thermal load at higher speeds in the absence of any cooling medium. Excessive heat accelerates flank wear and promotes built-up edge formation on the rake face. The BUE fractures intermittently and deposits irregularly on the machined surface, directly raising Ra values. Under vortex tube cooling, the two speed curves remained closely spaced across most of the feed range. Only a marginal divergence appeared at f = 0.12 mm/rev. The cold air jet generated by the Ranque–Hilsch effect provided sufficient convective cooling to offset the thermal damage that higher speed would otherwise impose. As a result, cutting speed had a limited influence on Ra in this environment. In the NMQL condition the effect of cutting speed was the least pronounced among all three environments. The Ra curves at *V_c_* = 30 and 60 m/min followed a nearly identical trajectory across the entire feed range. The pressurized lubricant mist effectively controlled both frictional heating and adhesion at the cutting interface, making the surface quality largely insensitive to the speed variation tested. This highlights the ability of NMQL to stabilize surface generation conditions across a range of cutting speeds.

Among the three environments, dry machining consistently yielded the highest Ra values throughout the entire parametric range. The complete absence of lubrication and cooling allows unrestricted friction at the tool–chip and tool–workpiece interfaces. This generates excessive heat, promotes adhesive wear, and encourages BUE formation. The combined action of these mechanisms produces irregular surface deposits and thermally induced texture defects, making dry machining the least favorable condition for surface quality. Vortex tube cooling reduced Ra appreciably compared with dry machining. The improvement is primarily attributed to the temperature reduction achieved by the cold air stream, which suppresses tool softening and limits BUE development. However, since the vortex tube delivers no lubricating film, the frictional component of surface degradation remains active. This limits the extent of improvement and explains why vortex tube performance, while better than dry machining, could not match that of NMQL. NMQL with MWCNT nanoparticles delivered the best surface finish at lower and intermediate feed rates. The fine mist of Eraoil KT/2000 forms a thin lubricating film at the tool–chip interface, reducing friction and suppressing material adhesion to the cutting edge [35]. The MWCNT nanoparticles further enhance this effect by possibly acting as nanoscale rolling elements between contacting asperities, reducing direct metallic contact and surface scoring [8]. Accordingly, these mechanisms are discussed here as plausible explanations based on the existing literature, while the primary experimental observation is that the MWCNT-NMQL system reduced surface roughness compared with dry machining under the tested conditions. Their high axial thermal conductivity may contribute to improved thermal transport and more stable surface generation [35]. Dry machining produced irregular chips, indicating unstable cutting, while NMQL and vortex cooling resulted in more uniform chips, reflecting stable conditions and better surface quality.

Table 6 presents the corresponding ANOVA results. The feed rate was identified as the most influential factor, accounting for 43.81% of the total variation, and its effect was statistically significant (*p* < 0.001). This finding is consistent with the experimental results, in which surface roughness generally increased with increasing feed rate because of the larger feed marks and greater undeformed chip thickness. The second-order component associated with the coded cutting condition was the second most influential statistical term, with a contribution ratio of 30.67%, and was statistically significant (*p* < 0.001). In addition, the first-order component associated with cutting condition contributed 11.66% and was also statistically significant (*p* = 0.006). Under the adopted −1, 0, and +1 coding, the first-order component primarily represents the contrast between the two outer coded environments, dry and NMQL, whereas the second-order component represents the deviation of the intermediate coded environment, vortex, from the response pattern of the two outer levels. Therefore, the significant second-order term should be interpreted as a statistical contrast among the three cutting environments rather than as evidence of a physically nonlinear relationship between cutting environment and surface roughness. These statistical findings are consistent with the experimental observations, where NMQL generally produced the lowest surface roughness values, dry machining resulted in the highest values, and vortex tube cooling provided intermediate surface finish performance. Cutting speed had only a minor contribution of 0.20% and was statistically non-significant (*p* = 0.654), indicating that its effect on surface roughness was limited within the investigated range. Similarly, the second-order feed term and all two-way interaction terms were statistically non-significant (*p* > 0.05). The error contribution was 8.16%, showing that the ANOVA model explained approximately 91.84% of the total variability in the surface roughness data. Overall, the results indicate that the feed rate was the dominant parameter affecting surface roughness, while the cutting environment also played an important role, as demonstrated by the statistically significant first- and second-order contrasts among the dry, vortex, and NMQL conditions.

### 3.4. Chip Morphology

**Figure 5:**
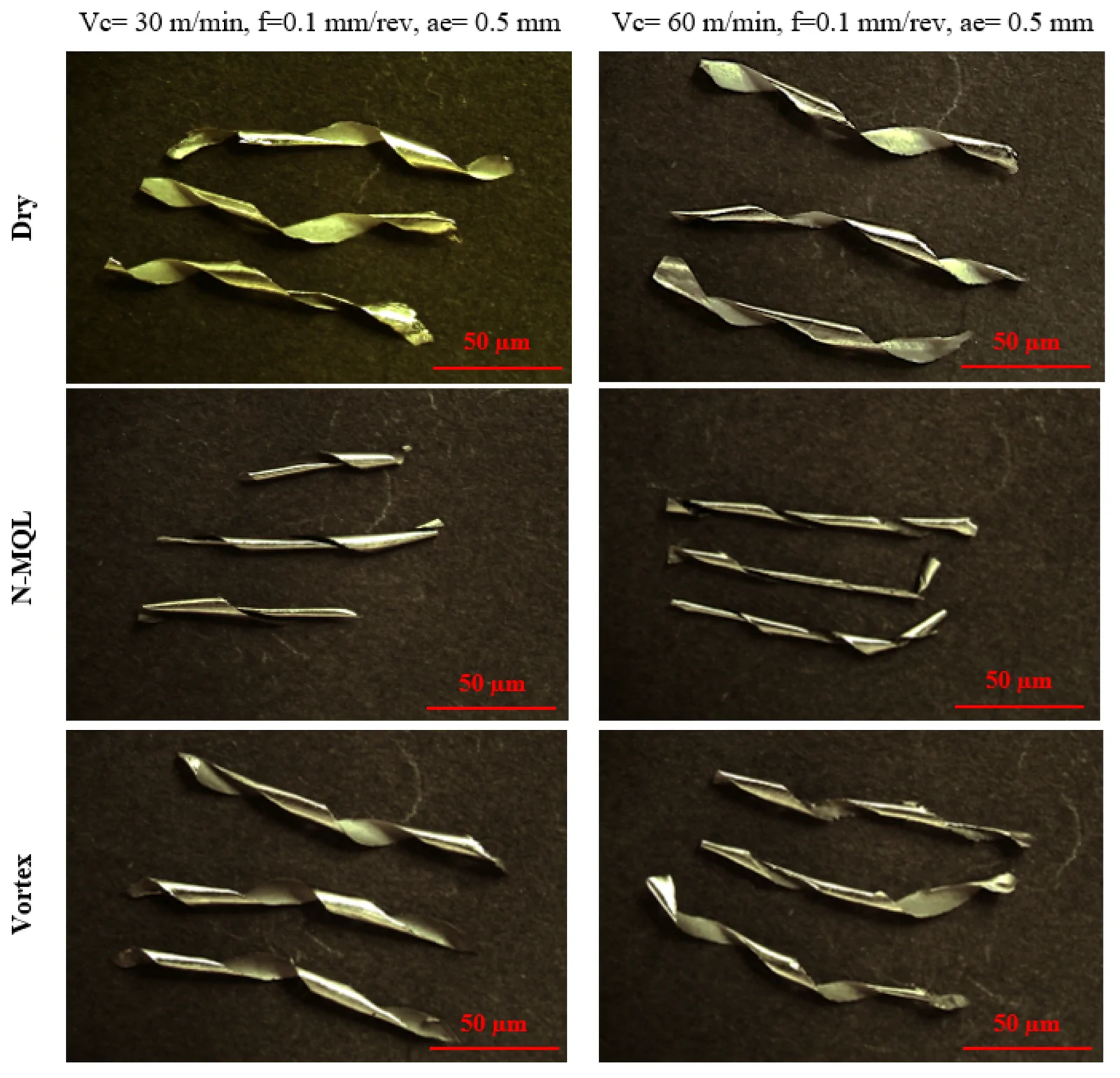
Chip morphology of Ti6Al7Nb after side milling under dry, NMQL, and vortex tube cooling conditions at Vc = 30 and 60 m/min and f = 0.10 mm/rev.

The morphology of chips is one of the important factors that influence the thermo-mechanical behavior of the cutting process, especially in titanium alloys, due to its low thermal conductivity, which makes heat dissipation from the cutting zone limited, resulting in localized plastic deformation. Therefore, cutting temperature, tool–chip friction, adhesion and material flow stability on the rake face play a significant role in chip formation in Ti6Al7Nb. The chip samples were viewed with an Mshot microscope. Figure 5 illustrates the chip morphology obtained while side milling Ti6Al7Nb under dry, NMQL and vortex tube cooling conditions at a feed rate of 0.10 mm/rev and cutting speeds of 30 and 60 m/min. The chips produced by dry machining were very curved, twisted and irregular in shape at both cutting speeds. The non-uniform curvature and the ribbon formation in the case of dry chips were observed at Vc = 30 m/min, which is a sign of high friction and intermittent chip flow at the tool–chip interface. There was no external cooling or lubrication; the plastic deformation and rubbing resulted in the generation of heat, which was concentrated near the cutting edge. This condition resulted in an adhesion between the chip and the rake face, which increased the resistance to the sliding of the chips and caused the chips to curl out of shape. The high temperature loadings and stronger shear localization seemed to increase with an increase in Vc (at 60 m/min, the dry chip was more severely deformed and compacted). The increased cutting speed resulted in higher sliding velocity at the tool–chip interface, leading to higher heat generation and formation of segmented chips. This observation is in line with the previously reported result with dry cutting, which was shown to yield the highest cutting temperature, cutting force and surface roughness in the dry machining experiments.

The chips in the NMQL looked more continuous, uniform and controlled than in dry machining. The shape of the chip was more curled with less random twisting at Vc = 30 m/min, indicating better chip evacuation and friction over the rake face. This has been attributed primarily to the lubricating effect of the Eraoil KT/2000 oil mist, which creates a thin film between the tool and chip. The suspended MWCNT nanoparticles may further enhance the tribological characteristics by reducing direct asperity contact and help to act as rolling or sliding elements in the interface as nanoscale elements. As such, chip flow is smoother, adhesion is reduced, and the likelihood of creating severe chip distortion is decreased. Even for the NMQL chip, curling and segmentation were observed at Vc = 60 m/min, but the morphology was more stable than the morphology of the corresponding dry chip. This verifies that NMQL can effectively control the frictional and thermal situation even at a higher cutting speed. These observations are corroborated by the cutting force and surface roughness results, which gave the best surface finish and least resultant force with NMQL.

The cooling condition in the vortex tube cutting also produced better morphology of the chips than dry cutting. The cold air jet resulted in a more regular ribbon-like chip at Vc = 30 m/min than the dry chip, suggesting the cold air jet had the ability to minimize the excessive accumulation of heat in the cutting zone. The cooling mechanism of the vortex tube is primarily thermal instead of tribological. Consequently, it can effectively reduce the cutting temperature by forced convection but cannot form a lubricating film at the tool–chip interface. Due to this, the vortex-cooled chips were more stable than the dry chips and showed some level of control, but less than the dry chips produced under NMQL. Even though the severe thermal distortion was suppressed at Vc = 60 m/min, the severe frictional contact along the rake face and absence of lubrication still caused noticeable curling of the chip. As is seen from the comparison of the cutting speeds, the chip deformation increased in all environments while increasing the cutting speed from *V*c = 30 m/min to Vc = 60 m/min. This is not unexpected, as an increase in cutting speed increases the strain rate and heat production in the primary and secondary deformation zones. The generated heat cannot easily be transported into the workpiece, which is a low thermal conductivity material, and thus leads to thermal softening and adiabatic shear localization. This leads to a serrated or segmented nature of the chips that is often seen when machining titanium alloys. This effect, however, was strongly influenced by the cutting environment. The most stable chip morphology was observed for dry machining, the most controlled for NMQL, and the most favorable for vortex cooling, with the largest friction reduction at the same time. In general, the chip morphology results were confirmed as above, which is the same as the cutting temperature, cutting force and surface roughness results. High heat concentration, adhesion and friction caused irregular and very deformed chips during dry machining. NMQL showed the best chip morphology due to both the lubrication and heat transfer effects, resulting in better chip flow and less increased severity of tool–chip interaction. The thermal damage reduction and the improvement in the chip formation rate were shown to be effective in vortex tube cooling compared with the dry cooling process but could not be sufficient to control the friction-induced chip deformations in the absence of lubrication. Hence, the morphological analysis of chips confirms that NMQL is more effective for tribological improvement, and vortex tube cooling is more effective for thermal control during side milling of Ti6Al7Nb.

### 3.5. Tool Life and Tool Wear Behavior

**Figure 6:**
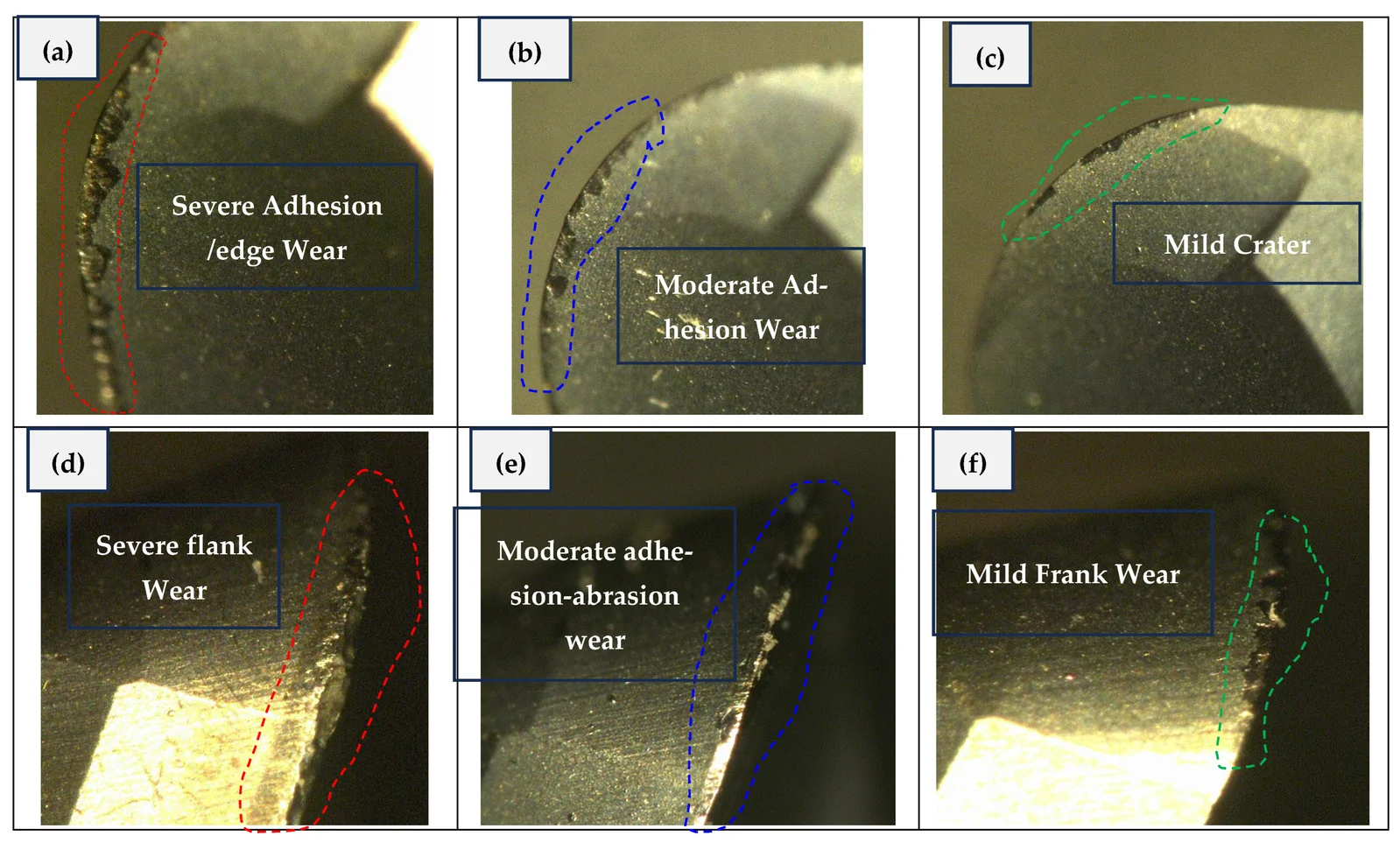
Rake and flank wear morphology of AlCrN-coated carbide end mill after side milling of Ti6Al7Nb at Vc = 60 m/min and f = 0.12 mm/rev: (**a**,**d**) dry, (**b**,**e**) N-MQL, and (**c**,**f**) vortex tube cooling.

Figure 6 shows the rake and flank wear morphology of the Karcan 155508010 Ø8 X-PER AlCrN-coated carbide tool after machining Ti6Al7Nb under the severe cutting condition of Vc = 60 m/min and f = 0.12 mm/rev. In dry machining, Figure 6a shows clear rake face damage, including adhered workpiece material, edge darkening, and local chipping near the cutting edge. This indicates strong tool–chip adhesion, high interface temperature, and possible coating degradation. The corresponding flank surface in Figure 6d also shows severe wear marks and irregular edge damage. The absence of cooling and lubrication promotes high friction, heat accumulation, built-up edge formation, abrasive scratching, and adhesive wear, making dry cutting the worst condition.

Under N-MQL, Figure 6b shows lower rake face damage than dry cutting, with reduced adhesion and a comparatively cleaner cutting edge. The improved wear behavior under N-MQL may be associated with the lubricating action of the base oil mist together with possible MWCNT-assisted mechanisms, such as tribofilm formation and rolling/sliding effects reported in previous studies. However, because conventional MQL was not separately evaluated, these nanoparticle-specific mechanisms cannot be independently confirmed from the present experiments. However, some adhered particles and crater-like marks are still visible, suggesting that lubrication alone could not fully suppress thermal softening and chemical/adhesive interaction at this high cutting speed and feed. Similarly, Figure 6e shows moderate flank wear with visible abrasion and local adhesion. Therefore, N-MQL provided the second-best wear resistance.

The best wear condition was observed under vortex tube cooling, as shown in Figure 6c,f. The rake face in Figure 6c appears smoother and cleaner, with less adhered material and lower edge deterioration compared with both dry and N-MQL conditions. The flank surface in Figure 6f also shows limited flank wear and relatively stable cutting-edge geometry. This improvement is mainly attributed to the excellent cooling capacity of the vortex tube, which reduces cutting zone temperature, thermal softening of the AlCrN coating/substrate, and adhesion of Ti6Al7Nb to the tool surface. The cold air jet may also assist chip evacuation and produce a minor anti-adhesion or Leidenfrost-like interfacial effect, reducing direct sticking between the chip and rake face.

**Figure 7:**
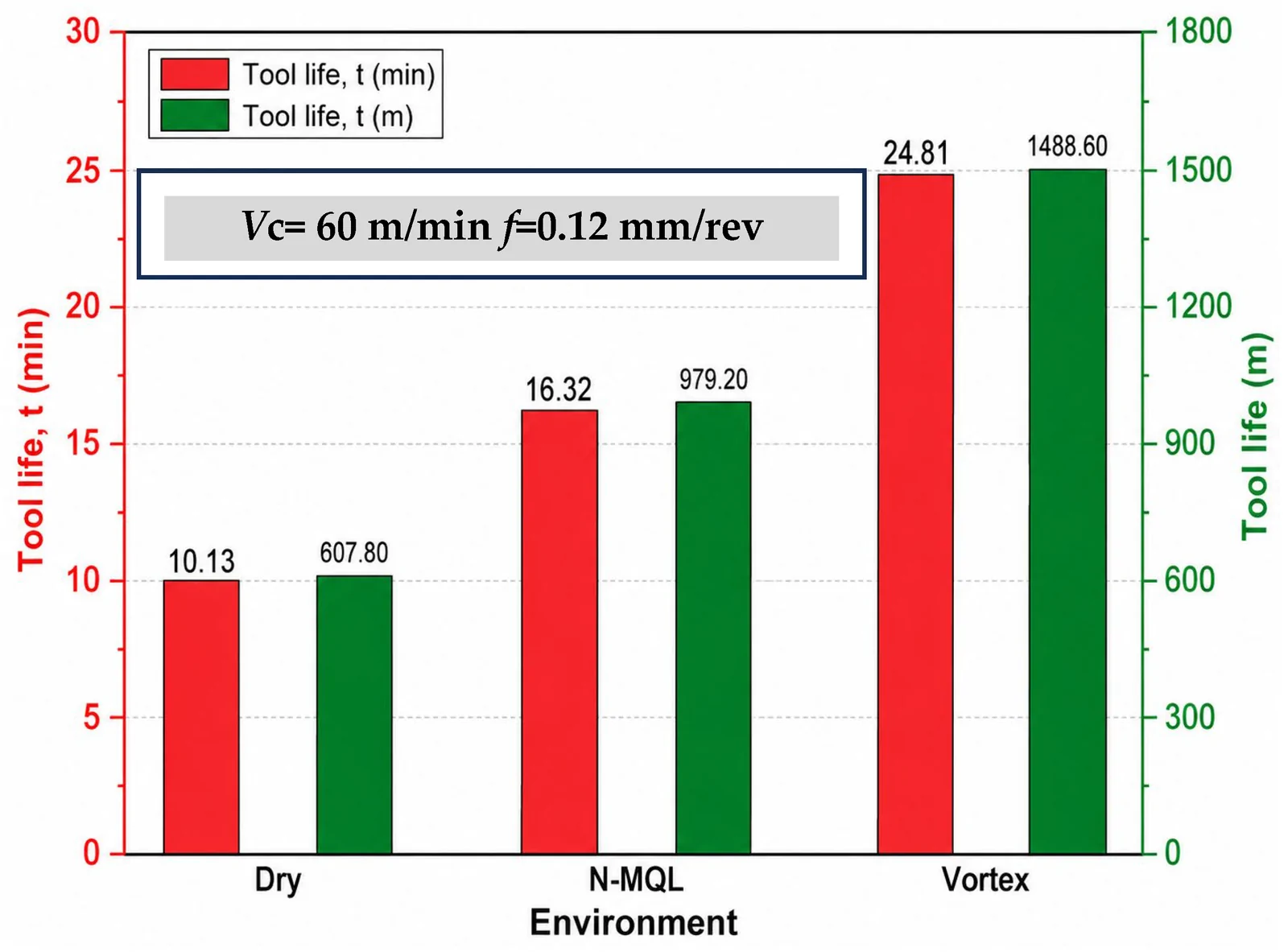
Tool life of Ti6Al7Nb under Vc = 60 m/min and f = 0.12 mm/rev.

Figure 7 presents the tool life response obtained under dry, N-MQL, and vortex tube cooling environments at Vc = 60 m/min and f = 0.12 mm/rev. Tool life was determined based on the experimentally observed tool wear progression, where the end of tool life was defined when the flank wear approached the commonly used failure criterion of approximately 300 μm. Since wear was measured at discrete machining intervals, interpolation and, where necessary, limited extrapolation were used to estimate the corresponding tool life values.

The tool life increased from 10.13 min in dry machining to 16.32 min under N-MQL and reached the maximum value of 24.81 min under vortex cooling. A similar trend was observed for the cutting length-based tool life, increasing from 607.80 m for dry machining to 979.20 m for N-MQL and 1488.60 m for vortex cooling. These results are consistent with the wear morphologies shown in Figure 6, where dry machining exhibited severe rake and flank wear due to high friction, thermal loading, adhesion, and edge deterioration. N-MQL reduced tool wear by improving lubrication, lowering the coefficient of friction, and suppressing adhesion at the tool–chip interface; however, some flank and crater wear still remained due to the severe cutting condition. Vortex cooling provided the best tool life performance because its strong cooling effect reduced cutting zone temperature, thermal softening, adhesion, and coating degradation, while also supporting chip removal and minor anti-adhesion effects.

### 3.6. Optimization Results

Multi-criteria decision making (MCDM) techniques were used to investigate the best cutting parameters for side milling Ti6Al7Nb. The combined effect of cutting temperature, resultant cutting force, and surface roughness was evaluated by three techniques: CoCoSo (combined compromise solution), TOPSIS (technique for order of preference by similarity to ideal solution), and MOORA (multi-objective optimization on the basis of ratio analysis). These methods were selected because TOPSIS evaluates alternatives according to their proximity to the ideal solution, CoCoSo provides a compromise ranking through multiple aggregation strategies, and the MOORA ratio system and reference point approaches provide complementary ratio- and reference-based evaluations. Their combined use allows the consistency and robustness of the ranking to be cross-checked using different decision-making principles. All responses were given equal weight (0.333) because each plays an important role in biomedical machining, including thermal control, tool load, and surface quality. This approach was adopted to evaluate the overall machinability performance without giving subjective priority to any single response. Since the study aimed to compare the cutting environments under a balanced performance perspective rather than an application-specific preference, additional sensitivity analysis was not considered necessary. Furthermore, the use of different MCDM methods allowed the consistency of the ranking results to be checked comparatively, and the best performing alternatives showed good agreement among the applied methods. For the CoCoSo method that combines WASPAS, SAW, and EWP methods, the decision matrix was normalized, and the performance scores were calculated with three aggregation strategies. The overall machinability of the experiments was ranked as follows: after vortex cooling, Experiment 3 (Vc = 30 m/min, f = 0.08 mm/rev) and after NMQL, Experiment 2 (Vc = 30 m/min, f = 0.08 mm/rev). The results indicate that the vortex cooling has beneficial effects on the reduction in temperatures in the cutting zone, the control of chip formation, and the minimization of thermal softening of the material, whereas the other beneficial effect of NMQL is mainly associated with lubrication and the nanoscale rolling effect of MWCNTs on the surface of the part. The results of the experimental measurements of temperature, force and surface roughness are compared with the results of the CoCoSo, which show that the method is able to represent the combined effect of thermal and tribological phenomena on the machinability. The TOPSIS method, which is based on the Euclidean distance of the alternatives from ideal and anti-ideal solutions, also ranked Experiment 3 as the best and Experiment 2 as the second best condition. When using TOPSIS, the closeness of each condition to the theoretical optimum, with minimum temperature, force and surface roughness, is highlighted quantitatively. The method is useful to emphasize the effective cooling ability of the vortex developed at low speed, which removes heat from the tool–chip interface, prevents any thermal softening of the chips, and ensures the stable chip flow. When compared with NMQL, the benefits of low friction, adhesion, and good surface finish are generally attractive; however, the cooling ability is somewhat less desirable, and this is the only drawback that arises if temperature is considered as part of a multi-response evaluation. The strength of the optimum obtained from TOPSIS and CoCoSo validates the reliability of the optimum. These results were confirmed by the application of the MOORA method with both the ratio system and the reference point approach. Both the ratio system and the reference point approach involve calculating a normalized and weighted score for each performance and then aggregating these scores for the ratio system and calculating the distance between each alternative and the ideal values for the reference point approach. In both cases, Experiment 3 came out with the top score and Experiment 2 with the second. Only minor differences in the lower ranked experiments were observed, which indicates that the higher ranked alternatives were independent of the computational method. MOORA is designed to reflect the collective influence of all three of these factors, namely thermal management (vortex cooling), mechanical load reduction (low feed rate), and friction control (NMQL), on overall machinability.

Table 7 gives a comparative summary of all 18 experiments of the CoCoSo method, the TOPSIS method, and the MOORA method. As can be seen from Experiment 3, the highest rank was obtained when using vortex cooling at a low speed and a low feed rate, thus showing that the multi-response performance of vortex cooling at a low speed and low feed rate is the best, followed closely by NMQL at the same parameters, especially for the cutting force reduction and surface finish improvement. Dry machining was invariably the worst as it produced high temperature build-up, friction, and irregular surfaces.

A weight sensitivity analysis was also conducted due to the possibility of weighting the responses that may alter the MCDM ranking, as shown in Table 8. Three priority cases were also taken into account: temperature-dominant weighting, force-dominant weighting, and roughness-dominant weighting. This analysis was applied to see if an optimum condition changed when the thermal control, mechanical loading, or surface quality was given the most importance. The equal weight ranking thus gives an overall indication of the condition, and the sensitivity analysis offers guidance for the best choice of machining parameters based on the application-specific priority.

## 4. Conclusions

The current study examined the side milling performance of biomedical titanium alloy (Ti6Al7Nb) under three different cooling conditions: dry, MWCNT-based NMQL, and vortex tube cooling. The cutting experiments were carried out at cutting speeds of 30 and 60 m/min and the feed rates of 0.08, 0.10, and 0.12 mm/rev with constant axial and radial depths of cut of 1 mm and 0.5 mm, respectively. The main conclusions are as follows:

(1) Under all cutting conditions, the cutting parameters of cutting speed and feed rate were observed to increase the cutting temperature. The lowest temperatures were obtained with vortex tube cooling, with temperatures of approximately 100–116 °C at 30 m/min and 130–150 °C at 60 m/min, which is 25–36% lower than the temperatures obtained with dry machining.

(2) The MWCNT-based NMQL condition produced the lowest cutting force, with a reduction of about 7–28% compared with dry machining. This reflects the overall effect of the NMQL system (base oil mist plus possible nanoparticle effects). Since conventional MQL without MWCNTs was not tested, the specific contribution of MWCNTs cannot be separated. Possible mechanisms such as tribofilm formation, rolling/sliding, and improved heat transfer are suggested based on the literature, not directly proven here.

(3) In general, the surface roughness increased with the feed rate; it was found that the feed rate had more impact on the surface quality than the cutting speed. The surface finish achieved was best with NMQL, with a reduction in the Ra of some 10–20% to that achieved for the dry machining process, because there is less friction, adhesion, and surface damage with NMQL.

(4) Chip morphology corroborated the measured force, temperature, and roughness results. For dry machining, the chips were more irregular and highly deformed, while the chips obtained from NMQL were more regular and uniform. Vortex cooling showed less thermal deformation and better chip forming than dry cutting.

(5) At Vc = 60 m/min and f = 0.12 mm/rev, tool life increased from 10.13 min under dry machining to 16.32 min under N-MQL and 24.81 min under vortex cooling. Thus, N-MQL provided approximately 1.61 times the dry cutting tool life, whereas vortex cooling extended it to approximately 2.45 times the dry cutting value.

The results of all four methods (TOPSIS, CoCoSo, MOORA ratio system, and MOORA reference point approach) showed that Experiment 2 (NMQL) was the second best choice, with Experiment 3 (vortex cooling) being the optimum condition. Based on this study, the use of vortex tube cooling is suggested if only thermal control and tool life extension is desired, and the use of MWCNT-based NMQL is suggested if cutting force reduction and surface finish improvement are desired. The overall performance of the dry machining process is the worst and should not be applied to precision side milling of Ti6Al7Nb biomedical alloy.

## Figures and Tables

**Table 1:** Comparison of the present study with related titanium machining studies.

Study	Material	Process	Cooling/Lubrication	Main Responses	Limitation/Gap
Sun et al. [8]	Ti6Al7Nb	Cryogenic machining	Cryogenic/dry/flood	Surface quality	Not MWCNT-NMQL or vortex side milling
An et al. [11]	Ti6Al4V	Side milling	Dry/scCO_2_ + MQL	Tool wear/surface	Different alloy; no Ti6Al7Nb
Hegab et al. [25]	Ti6Al4V	Machining	MWCNT-MQL	Force/roughness	Turning, not side milling Ti6Al7Nb
Aydın et al. [8]	Ti6Al4V	Milling	Nanofluid MQL	Surface/sustainability	Different alloy; no vortex comparison
Present study	Ti6Al7Nb	Side milling	Dry, vortex, MWCNT-NMQL	Temperature, force, Ra, chip morphology, MCDM	Direct comparison under identical side milling conditions

**Table 2:** Experimental parameters and cutting conditions for Ti6Al7Nb milling trials.

Exp #	Cutting Speed (m/min)	Cutting Speed (rpm)	Feed Rate (mm/rev)	Cutting Condition
1	30	1194	0.08	Dry
2	30	1194	0.08	NMQL
3	30	1194	0.08	Vortex
4	30	1194	0.1	Dry
5	30	1194	0.1	NMQL
6	30	1194	0.1	Vortex
7	30	1194	0.12	Dry
8	30	1194	0.12	NMQL
9	30	1194	0.12	Vortex
10	60	2389	0.08	Dry
11	60	2389	0.08	NMQL
12	60	2389	0.08	Vortex
13	60	2389	0.1	Dry
14	60	2389	0.1	NMQL
15	60	2389	0.1	Vortex
16	60	2389	0.12	Dry
17	60	2389	0.12	NMQL
18	60	2389	0.12	Vortex

**Table 3:** Process parameters evaluated under the selected machining conditions.

Parameters	Description
Machine tool	First MCV-300 CNC milling machine.
Cutting tool	Karcan 155508010 Ø8 X-PER AlCrN-coated WC end mill.
Workpiece material	Ti6Al7Nb—36 × 2.5 mm.
Cutting parameters	Cutting speed *V_c_* (m/min): 30, 60. Feed rate *f* (mm/rev): 0.08, 0.1, 0.12. Depth of cut (mm): 1. Radial depth of cut (mm): 0.5. Cutting length (mm): 14.
Environments	Dry, NMQL, vortex tube.
NMQL lubricant	Eraoil KT/2000. Nano particles: MWCNT by 0.2%.
	Flow rate: 90 mL/h. Nozzle diameter: 1 mm. Nozzle angle: 40°. Nozzle to cut distance: 50 mm.
Vortex tube	Air pressure: 6 bar. Nozzle dimension: 10 × 2 mm rectangular. Nozzle to cut distance: 30 mm.

**Table 4:** Full quadratic ANOVA results of cutting temperature.

Source	Seq SS	Contribution	F-Value	*p*-Value	Remarks
Cutting Speed	3584.2	25.76%	843.35	<0.001	Significant
Feed	1240.3	8.92%	291.84	<0.001	Significant
Cutting Condition	9020.1	64.83%	2122.37	<0.001	Significant
Feed × Feed	0.1	0.00%	0.03	0.875	Non-Significant
Cutting Condition × Cutting Condition	1.4	0.01%	0.32	0.585	Non-Significant
Cutting Speed × Feed	1.3	0.01%	0.31	0.589	Non-Significant
Cutting Speed × Cutting Condition	18.8	0.13%	4.41	0.065	Non-Significant
Feed × Cutting Condition	8	0.06%	1.88	0.203	Non-Significant
Error	38.3	0.27%			
Total	13,912.4	100.00%			

**Table 5:** Full quadratic ANOVA results of resultant cutting force.

Source	Seq SS	Contribution	F-Value	*p*-Value	Remarks
Cutting Speed	0.3	0.00%	0	0.969	Non-Significant
Feed	8338	52.76%	48.39	<0.001	Significant
Cutting Condition	72.8	0.46%	0.42	0.532	Non-Significant
Feed × Feed	16.4	0.10%	0.09	0.765	Non-Significant
Cutting Condition × Cutting Condition	5481.6	34.68%	31.81	<0.001	Significant
Cutting Speed × Feed	28.6	0.18%	0.17	0.693	Non-Significant
Cutting Speed × Cutting Condition	13.1	0.08%	0.08	0.789	Non-Significant
Feed × Cutting Condition	303.6	1.92%	1.76	0.217	Non-Significant
Error	1550.9	9.81%			
Total	15,805.2	100.00%			

**Table 6:** Full quadratic ANOVA results of surface roughness.

Source	Seq SS	Contribution	F-Value	*p*-Value	Remarks
Cutting Speed	0.000012	0.20%	0.22	0.654	Non-Significant
Feed	0.002789	43.81%	48.35	<0.001	Significant
Cutting Condition	0.000742	11.66%	12.86	0.006	Significant
Feed × Feed	0.000163	2.57%	2.83	0.127	Non-Significant
Cutting Condition × Cutting Condition	0.001952	30.67%	33.84	<0.001	Significant
Cutting Speed × Feed	0.000065	1.02%	1.12	0.317	Non-Significant
Cutting Speed × Cutting Condition	0.000014	0.22%	0.24	0.635	Non-Significant
Feed × Cutting Condition	0.000109	1.71%	1.88	0.203	Non-Significant
Error	0.000519	8.16%			
Total	0.006365	100.00%			

**Table 7:** Comparison of multi-criteria decision-making methods.

Exp. No	Cutting Conditions	TOPSIS	COCOSO	MOORA Ratio	MOORA Ref. Point
1	Dry	8	7	8	8
2	NMQL	2	2	2	2
3	Vortex	1	1	1	1
4	Dry	15	14	14	11
5	NMQL	3	4	4	3
6	Vortex	6	8	5	10
7	Dry	17	17	17	15
8	NMQL	11	10	11	6
9	Vortex	9	12	10	9
10	Dry	13	11	12	16
11	NMQL	4	3	3	7
12	Vortex	5	6	6	5
13	Dry	17	16	16	17
14	NMQL	10	5	7	11
15	Vortex	7	9	9	4
16	Dry	18	18	18	18
17	NMQL	12	13	13	14
18	Vortex	14	15	15	13

**Table 8:** Weight sensitivity analysis of MCDM ranking under different response priority scenarios for Ti6Al7Nb side milling.

Scenario	Temperature Weight	Force Weight	Ra Weight	Best Condition	Interpretation
Equal weight	0.333	0.333	0.333	Vortex, 30, 0.08	Balanced performance
Temperature priority	0.60	0.20	0.20	Vortex, 30, 0.08	Thermal control
Force priority	0.20	0.60	0.20	NMQL, 30/60, 0.08	Mechanical load
Roughness priority	0.20	0.20	0.60	NMQL, 30/60, 0.08	Surface finish

## Data Availability

The original contributions presented in this study are included in the article. Further inquiries can be directed to the corresponding authors.
